# Acknowledgement to Reviewers of *Pharmaceuticals* in 2017

**DOI:** 10.3390/ph11010005

**Published:** 2018-01-10

**Authors:** Pharmaceuticals Editorial Office

**Affiliations:** MDPI AG, St. Alban-Anlage 66, 4052 Basel, Switzerland

Peer review is an essential part in the publication process, ensuring that *Pharmaceuticals* maintains high quality standards for its published papers. In 2017, a total of 97 papers were published in the journal. Thanks to the cooperation of our reviewers, the median time to first decision was 24 days and the median time to publication was 47 days. The editors would like to express their sincere gratitude to the following reviewers for their time and dedication in 2017:
Adams, JamesLibrowski, TadeuszAdriaenssens, Evelien M.Lin, BorenAlves, Isabel D.Lin, XiaojieAmbrosini, ValentinaLinder, StigAmetamey, Simon MensahLinhardt, RobertAmpofo, EmmanuelLitchfield, DavidAnderson, CarolynLiu, YuArgia, KatsuhikoLiu, StanleyArsenyadis, StellyosLiu, Hai-yangAsai, DaisukeLiu, Jun-JenAxelsson, AndersLollo, GiovannaBalcão, Victor M.López-Abán, JulioBarraud, NicolasLovell, JonathanBar-Shalom, DanielLudwiczuk, AgnieszkaBenoist, EricMalik, DanishBertini, SabrinaMalinowska, BarbaraBiagini, GiuseppeMarchand, PascalBidwai, AshokMaruo, KohjiBoag, Peter R.Matthews, SusanBotta, BrunoMatuszak, ZenonBrooks, Amanda E.Maurer, AndreasCaffrey, Conor R.Mayence, AnnieCai, ZhengxinMcHugh, PatrickCapasso, RaffaeleMedina, DiegoCaputo, Gregory A.Meegan, Mary JaneCascini, Giuseppe LucioMehta, AmitkumarCastagnolo, DanieleMindt, Thomas L.Castñeiras, AlfonsoMinghetti, PaolaCaudle, Kelly EMiranda, KatrinaCeranowicz, PiotrMiyata, YoshihikoČeřovský, VáclavModos, DezsöChaud, Marco V.Mohr, Justin T.Chen, QiushuiMomparler, RichardCheong, AlexMonclus, MichelChiarelli, LaurentMontenarh, MathiasCho, Cheong-WeonMoreno, J.J.Chou, JoshuaMorgat, ClémentChubanov, VladimirMori, YasuoChun, Heung JaeNair, DevathaChung, DonghoonNamasivayam, VigneshwaranCole-Hamilton, David J.Neumann, TerrenceColeman, MathewNewton, AlexandraCollins, Stuart G.Niinivehmas, SannaConnors, NealNilsson, AndersConstance, Jonathan E.Nisnevitch, MarinaCoote, PeterNorris, JamesCopolovici, Dana MariaOtvos, LaszloCornell, Robert A.Ozawa, ShogoCosta, PatriciaPaci, MaurizioCostantino, LucaPan, YangangCozza, GiorgioPan, JianboCzaplewski, CezaryPanaro, Maria AntoniettaDaverey, AmitaPark, Steven I.De Brevern, AlexandrePark, MaiyonDealwis, Chris G.Pascarelli, Nicola AntonioDi Franco, SimonePasqualoto, KerlyDoi, HisashiPeifer, ChristianDorado, PedroPeitzsch, ClaudiaDovat, SinisaPellei, MauraDussor, GregPerepelyuk, MarynaFang, MarongPietsch, MarkusFerris, WilliamPissarek, MargitFlashman, EmilyPletzer, DanielFleig, AndreaPoltronieri, PalmiroFlieger, JolantaPomin, VitorFormato, MarilenaPopa-Wagner, AurelForssell-Aronsson, EvaPorcheddu, AndreaFoster, Rebecca R.Poznański, JarosławFranken, NicolaasPrasad, VikasFröhlich, EleonorePrevette, Lisa E.Furukawa, TatsuhikoRabanal, FrancescGangloff, Sophie C.Raje, MithunGarrod, DavidRoder, ChristineGehrmann, MathiasSagan, SandrineGemmill, TrentSantoni, GiorgioGeng, FengSchibli, RogerGonzález-Muñiz, RosarioSchillaci, OrazioGorski, AndrzejSchwinté, PascaleGrande, FedoraSeidler, Daniela G.Guerrini, MarcoSelan, LauraGuillet, Benjamin AlainSetzer, William N.Guler, Mustafa O.Siegel, DionicioHahn, UlrichSilva, Nuno A.Hanna, AmgadSima, JianHardy, John G.Simitzis, PanayiotisHarris, EvaSizochenko, NataliaHaug, AlexanderSpeich, BenjaminHendrikse, HarrySrivenugopal, KalkunteHirao, IchiroStancu, Izabela-CristinaHomma, Miwako KatoStephens, Gary J.Hu, ShanghsiuSurguchov, AndreiHusain, MatloobTaghibiglou, ChangizIannazzo, DanielaTakano, KatsuraImai, ToshioThirstrup, KennethInokuchi, TsutomuThordarson, PallItarte, EmilioThorek, Daniel L. J.Jain, PrashantTimmerman, PeterJansen, GerritTing, Richard C. H.Jayasinghe, Suwan N.Tiwari, Rakesh KumarJiménez-Jiménez, Félix JavierToyofuku, MasanoriJohnson, Abbie CTrembley, Janeen HKaneko, ShingoTseng, Boo ShanKarmakar, ParthaTsurkan, Mikhail V.Kevadiya, BhaveahTurnbull, Jeremy EKhan, Faruk M.O.Vaidyanathan, GanesanKhang, GilsonValomon, AmandineKibble, MillaVan Damme, JoKinoshita, TakayoshiVergara, DanieleKintzios, SpyridonViggiano, AndreaKocoglu, MehmetVisentin, MicheleKontopidis, GeorgeVoliani, ValerioKostiainen, Mauri A.Wadsak, WolfgangKovacs, RichardWang, XiaohongKrammer, FlorianWang, WentianKubala, LukášWang, JingKumar, KrishanWildemann, BrittKumar, AshutoshWilliams, Christopher C.Kung, Hank F.Winkler, HeinzKweon, Oh-KyeongWinkler, ChristophKwon, Tae-HyukWolf-Rainer, AbrahamLange, RogierWu, BinLauder, BobWu, Ming JieLauritano, DorinaXavier, Ana C.Lazarowski, AlbertoYin, JianLees, Watson J.You, LiangLexchin, JoelZamyatnin, Andrey A.Li, XiaoboZhang, ZhenbinLi, Xiang-GuoZheng, Fang

